# *miR-4428* and *miR-185-5p* as Key Modulators of Insulin Sensitivity and Glucose Homeostasis: Insights into Pathways and Therapeutic Potential in Type 2 Diabetes Mellitus

**DOI:** 10.3390/biology14040424

**Published:** 2025-04-15

**Authors:** Yanisa Rattanapan, Thitinat Duangchan, Thaveesak Sai-ong, Takol Chareonsirisuthigul

**Affiliations:** 1 Medical Technology, School of Allied Health Sciences, Walailak University, Nakhon Si Thammarat 80160, Thailand; yanisa.rt@wu.ac.th (Y.R.); thitinat.du@wu.ac.th (T.D.); 2 Hematology and Transfusion Science Research Center, Walailak University, Nakhon Si Thammarat 80160, Thailand; 3 School of Public Health, Walailak University, Nakhon Si Thammarat 80160, Thailand; thaveesaksaiong@gmail.com; 4 Department of Pathology, Faculty of Medicine Ramathibodi Hospital, Mahidol University, Bangkok 10400, Thailand

**Keywords:** *miR-4428*, *miR-185-5p*, insulin sensitivity, glucose homeostasis, type 2 diabetes mellitus

## Abstract

Type 2 Diabetes Mellitus (T2DM) is a chronic metabolic disorder characterized by insulin resistance and dysregulation of glucose metabolism. MicroRNAs (miRNAs) such as *miR-4428* and *miR-185-5p* play critical roles in post-transcriptional regulation of genes involved in these processes, but their specific contributions to T2DM pathogenesis remain unclear. Plasma samples from T2DM patients and non-diabetic controls were analyzed for *miR-4428* and *miR-185-5p* expression using microarray and bioinformatics tools. Target genes were predicted, and pathway enrichment analysis was performed to explore biological roles. Differential expression analysis revealed a 2.3-fold upregulation of *miR-4428* and a 14.4-fold downregulation of *miR-185-5p* in T2DM patients compared to controls. Predicted targets such as *ADAR*, *KLF9*, and *SOGA1* were linked to glucose metabolism and insulin signaling pathways. Enrichment analysis highlighted associations with neuronal signaling, chromatin remodeling, and metabolic regulation pathways. *miR-4428* and *miR-185-5p* regulate critical insulin sensitivity and glucose metabolism pathways, making them promising biomarkers and therapeutic targets for managing T2DM. Future studies should validate these findings experimentally to advance miRNA-based interventions for T2DM and its complications.

## Simple Summary

*miR-4428* and *miR-185-5p* show altered expression in T2DM, with 2.3-fold upregulation and 14.4-fold downregulation, respectively. These miRNAs target genes associated with insulin sensitivity and glucose metabolism, including *ADAR, KLF9*, and *SOGA1*. Enrichment analysis connects them to neuronal signaling and chromatin remodeling. They are potential biomarkers and therapeutic targets for T2DM, pending further validation.

## 1. Introduction

Type 2 Diabetes Mellitus (T2DM) is a prevalent metabolic disorder characterized by a combination of insulin resistance and impaired insulin secretion from pancreatic β-cells, leading to chronic hyperglycemia [1,2]. As the body becomes less efficient in regulating blood glucose levels, T2DM progressively impacts multiple organ systems, resulting in complications such as cardiovascular disease, neuropathy, nephropathy, and increased susceptibility to infections [3]. The pathogenesis of T2DM involves both a diminished ability of the pancreas to produce sufficient insulin and reduced insulin sensitivity in peripheral tissues, particularly muscle, adipose tissue, and liver [4]. Insulin resistance prevents cells from efficiently responding to insulin, resulting in persistent hyperglycemia and an increased demand for insulin production, which, over time, exhausts β-cells [5]. Lifestyle factors, including high-calorie diets, sedentary behavior, and obesity, are strongly linked to the development and progression of T2DM, while genetic predisposition and family history highlight the complex interplay between genetics, environment, and metabolic health [6,7].

MicroRNAs (miRNAs) have emerged as critical regulators of gene expression, playing significant roles in cellular processes relevant to T2DM, including metabolism and inflammation [8]. These small non-coding RNAs (~22 nucleotides) regulate gene expression by binding to the 3′ untranslated regions (UTRs) of target mRNAs, influencing their stability and translation [9]. This post-transcriptional regulation can significantly impact insulin secretion, insulin receptor signaling, and glucose metabolism, with several miRNAs identified as biomarkers of insulin sensitivity and resistance [10,11]. For instance, *miR-375, miR-29*, and *miR-103/107* have been implicated in pancreatic β-cell dysfunction, insulin resistance, and adipocyte differentiation, respectively [12].

The dysregulation of miRNAs can affect insulin signaling pathways, which involve downstream events such as the activation of phosphoinositide 3-kinase (PI3K) and Akt pathways, promoting glucose uptake and metabolic regulation [13,14]. Insulin resistance disrupts the efficiency of these pathways, impairing glucose absorption and perpetuating hyperglycemia. The involvement of miRNAs in these processes offers an opportunity to restore metabolic balance and improve insulin sensitivity [4].

Recent studies have highlighted *miR-4428* as an emerging regulator in various pathological conditions, including colon adenocarcinoma, cervical cancer, and osteoarthritis, through its interactions with key regulatory pathways [15,16,17]. For instance, *miR-4428* is sponged by lncRNA ACTA2-AS1 in colon adenocarcinoma, leading to increased apoptosis, whereas in cervical cancer and osteoarthritis, *miR-4428* downregulation promotes tumor growth and cartilage degradation. Although its precise role in insulin sensitivity in T2DM remains unclear, *miR-4428’s* regulatory potential suggests modulating its expression may significantly improve insulin signaling and address metabolic complications associated with T2DM.

In addition, *miR-185-5p* has gained attention for its potential role in regulating insulin sensitivity and glucose homeostasis, positioning it as a promising therapeutic candidate for T2DM. Known to manage oxidative stress, inflammation, and apoptosis, *miR-185-5p*’s complete regulatory mechanisms and therapeutic potential remain insufficiently explored [18,19,20]. Clarifying the functions of *miR-185-5p* in T2DM, mainly through identifying its target genes and molecular interactions using bioinformatics tools, could address critical gaps in current treatment strategies. This could pave the way for developing targeted therapies that enhance insulin action, reduce hyperglycemia, and slow the progression of T2DM and related complications.

This study aims to investigate the expression profiles of *miR-4428* and *miR-185-5p* in T2DM patients and identify their potential target genes using bioinformatics tools. Pathway enrichment analysis will be performed to elucidate the biological processes and molecular pathways through which these miRNAs regulate insulin sensitivity and glucose metabolism. By analyzing the network of gene connections and signaling pathways modulated by *miR-4428* and *miR-185-5p*, this study seeks to provide insights into potential therapeutic targets, facilitating the development of personalized and effective treatments for T2DM.

## 2. Materials and Methods

### 2.1. Sample Collection

A total of 20 plasma samples were collected, comprising 10 from individuals diagnosed with Type 2 Diabetes Mellitus (T2DM) and 10 from non-diabetic controls. Participants included in the T2DM group met specific inclusion criteria, including a fasting blood sugar (FBS) level of ≥126 mg/dL and a glycated hemoglobin (HbA1c) level of ≥6.5%. Detailed demographic data are summarized in Supplementary Table S1. Exclusion criteria for the study encompassed individuals with other forms of diabetes, such as gestational diabetes and Type 1 Diabetes Mellitus (T1DM). The study protocol was rigorously reviewed and approved by the Human Research Ethics Committee at Walailak University, Thailand (WUEC-23-235-01).

### 2.2. miRNA Extraction

miRNA extraction from the plasma samples was performed using the HiPure Serum miRNA Kit (Magen, Guangzhou, China) according to the manufacturer’s guidelines. The quantity and purity of the extracted miRNA were assessed using the NanoDrop™ One/OneC Microvolume UV-Vis Spectrophotometer (Thermo Fisher Scientific, Waltham, MA, USA) to confirm sample quality before proceeding with downstream analyses.

The purity and quantity of extracted miRNA were assessed using the NanoDrop™ One/OneC Spectrophotometer (Thermo Fisher Scientific) to ensure sample integrity. Samples with an A260/A280 ratio between 1.8 and 2.0 were considered suitable for downstream processing. Additionally, the RNA integrity was confirmed using an Agilent 2100 Bioanalyzer, with an RNA integrity number (RIN) threshold of ≥7 required to proceed with microarray analysis.

### 2.3. Microarray Analysis

The miRNA expression profiles in the plasma samples were analyzed using the GeneChip™ miRNA 4.0 Assay (Applied Biosystems, Carlsbad, CA, USA). The assay was conducted following the manufacturer’s protocol. After hybridization, the microarray data were processed and analyzed using the Transcriptome Analysis Console (TAC) Software 4.0.3.14 (Thermo Fisher Scientific, Waltham, MA, USA) to identify differentially expressed miRNAs between the T2DM and non-diabetic control groups.

### 2.4. miRNA Secondary Structure Prediction

To investigate the secondary structures *of miR-4428* and *miR-185*, miRTarBase (https://ngdc.cncb.ac.cn/databasecommons/database/id/167, accessed on 3 February 2025), a comprehensive database for miRNA-target interactions, was utilized. The secondary structure of *miR-4428* was retrieved from miRTarBase 2025, which provides detailed information regarding the structure and function of this miRNA. Similarly, the secondary structure of *miR-185* was also analyzed via miRTarBase 2025.

### 2.5. Target Prediction of miR-4428 and miR-185-5p

Predicted target genes for *miR-4428* and *miR-185-5p* were identified using the miRDB database (https://mirdb.org/mirdb/index.html, accessed on 3 February 2025). A list of the top 95–100 predicted targets was generated based on prediction scores. These targets were further analyzed to assess their potential roles in relevant biological processes.

Although multiple databases (TargetScan, miRTarBase, and miRDB) were evaluated for target prediction, miRDB was selected for the final analysis because it provided the most extensive and comprehensive coverage of predicted target genes for both *miR-4428* and *miR-185-5p*, facilitating a more thorough interpretation of their regulatory roles.

### 2.6. Network Analysis of miR-4428 and miR-185-5p Target Genes

A network diagram illustrated the interactions of *miR-4428* and *miR-185-5p* target genes within insulin signaling and glucose metabolism pathways. Gene sets were subjected to enrichment analysis using the Enrichr tool (https://maayanlab.cloud/enrichr-kg, accessed on 3 February 2025) to identify significant pathways associated with the predicted targets.

### 2.7. Enrichment Analysis of Biological Processes and Pathways

Enrichment analysis was conducted on the predicted target genes of *miR-4428* and *miR-185-5p* to identify overrepresented biological processes and pathways. Enrichment bar charts were generated using the Enrichr platform (https://maayanlab.cloud/enrichr-kg, accessed on 3 February 2025), visually representing the significant pathways in which *miR-4428* and *miR-185-5p* are involved.

### 2.8. Summary of Enrichment Analysis Results

The enrichment analysis results were compiled into a comprehensive table, offering an overview of the key biological processes and pathways enriched among *miR-4428* and *miR-185-5p* target genes. This summary highlights the potential roles of these enriched pathways in disease mechanisms. The analysis was conducted using the Enrichr Knowledge Graph (https://maayanlab.cloud/enrichr-kg, accessed on 3 February 2025).

### 2.9. Assessment of miR-4428 and miR-185-5p Expression Profiles Across Human Organs Using miRNA Tissue Atlas 2025

To assess the expression profiles of miR-4428 and miR-185-5p across various human organs, data from the miRNA Tissue Atlas 2025 were utilized (https://ccb-compute2.cs.uni-saarland.de/mirnatissueatlas_2025, accessed on 3 February 2025). The expression levels of miR-4428 by organ were obtained from the Atlas 2025 repository (https://ccb-compute2.cs.uni-saarland.de/mirnatissueatlas_2025/patterns/hsa/Atlas_2025_tissue/rpmm/mirna/hsa-miR-4428/yes/organ/no/, accessed on 3 February 2025). Similarly, the expression of miR-185-5p by organ was also retrieved from the same database (https://ccb-compute2.cs.uni-saarland.de/mirnatissueatlas_2025/patterns/hsa/Atlas_2025_tissue/rpmm/mirna/hsa-miR-185-5p/yes/organ/no/, accessed on 3 February 2025), which provides tissue-specific expression patterns for these miRNAs.

### 2.10. Disease Association Analysis of miR-4428 and miR-185

The miRBase database was used to investigate the disease associations of *miR-4428* and *miR-185*. The association of *miR-4428* with various human diseases was obtained from miRBase (https://www.mirbase.org/hairpin/MI0016767, accessed on 3 February 2025), which provides comprehensive information on miRNA sequences and their potential roles in human diseases. Similarly, the association of *miR-185* with human diseases was retrieved from miRBase (https://www.mirbase.org/hairpin/MI0000482, accessed on 3 February 2025), offering insights into its involvement in different pathological conditions.

### 2.11. Statistical Analysis

Statistical analysis was conducted using Transcriptome Analysis Console (TAC) 4.0.3.14 for microarray differential expression analysis. Enrichment analysis was performed using Enrichr, where *p*-values, false discovery rates (FDR), z-scores, and combined scores were calculated to determine the statistical significance of enriched pathways. The *p*-value represents the probability under the null hypothesis, the q-value is an FDR-adjusted *p*-value (Benjamini–Hochberg method), the z-score quantifies statistical significance relative to dataset distribution, and the combined score integrates both the *p*-value and z-score to rank enriched pathways.

## 3. Results

### 3.1. Microarray Analysis

**Figure 1:**
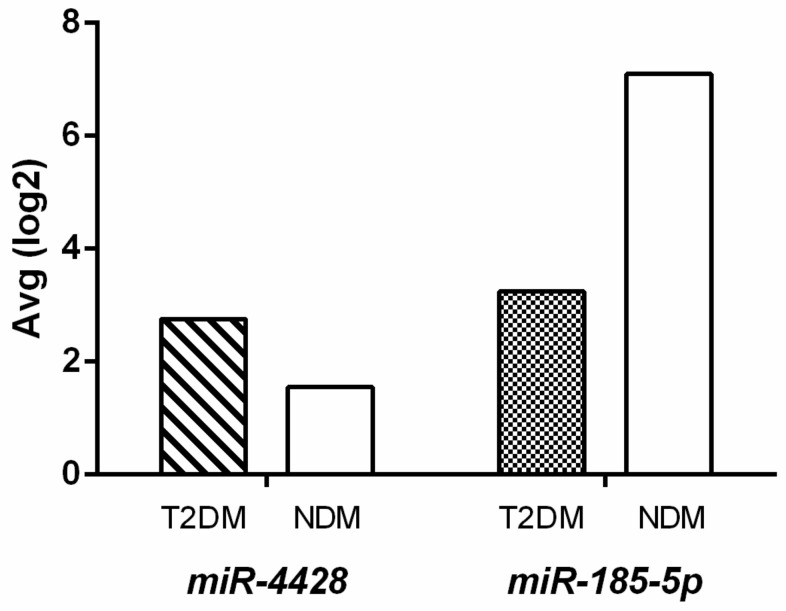
Differential expression of *miR-4428* and *miR-185-5p* in T2DM and NDM groups.

The differential expression analysis revealed significant modulation of *miR-4428* and *miR-185-5p* between Type 2 Diabetes Mellitus (T2DM) and non-diabetic (NDM) groups. *miR-4428* demonstrated a 2.3-fold upregulation in the T2DM group compared to the NDM group (DM Avg [log2] = 2.76; NDM Avg [log2] = 1.55) with a *p*-value of 0.003. However, its false discovery rate (FDR) *p*-value was 0.4167, indicating borderline statistical significance after correction for multiple testing. Conversely, *miR-185-5p* exhibited a 14.44-fold downregulation in the T2DM group (DM Avg [log2] = 3.25; NDM Avg [log2] = 7.1) with a *p*-value of 0.0072 and an FDR *p*-value of 0.5135, suggesting potential relevance despite not reaching stringent statistical thresholds (Figure 1).

### 3.2. miRNA Secondary Structure Prediction

**Figure 2:**
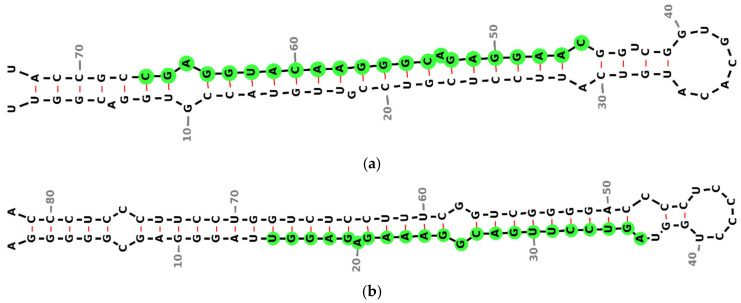
Predicted secondary structures of precursor miRNAs were analyzed in this study. (**a**) Precursor structure of *miR-4428*, located on chromosome 1 (chr1: 237471119–237471191), illustrating its characteristic stem–loop formation; (**b**) precursor structure of *miR-185*, located on chromosome 22 (chr22: 20033139–20033220), demonstrating a distinct stem–loop configuration. Green circles represent conserved nucleotides, black dashed lines indicate base-pairing interactions and red lines represent non-canonical base pairings.

The secondary structures of *miR-4428* and *miR-185* were analyzed and visualized to investigate their regulatory roles in insulin sensitivity and glucose homeostasis in T2DM. The precursor miRNA hsa-*mir-4428* exhibits a characteristic stem–loop structure with the sequence CAAGGAGACGGGAACAUGGAGC, localized on chromosome 1 (chr1: 237471119-237471191) (Figure 2a). Similarly, the precursor miRNA hsa-mir-185 forms a distinct stem–loop structure with the sequence UGGAGAGAAAGGCAGUUCCUGA, located on chromosome 22 (chr22: 20033139-20033220) (Figure 2b).

### 3.3. Target Prediction of miR-4428 and miR-185-5p

The top predicted targets for *miR-4428* and *miR-185-5p* were analyzed to identify their potential regulatory roles in insulin sensitivity and glucose homeostasis in T2DM. For *miR-4428*, the highest-ranked predicted targets include *EPHB1* (EPH receptor B1), *MECP2* (methyl-CpG binding protein 2), *KAT6A* (lysine acetyltransferase 6A), and *ADAR* (adenosine deaminase, RNA-specific), all with target scores above 98. Other notable targets include *RELN* (reelin), *CCT8* (chaperonin containing TCP1 subunit 8), and *KLF9* (Kruppel-like factor 9).

For *miR-185-5p*, the top predicted targets include *SMG7* (nonsense-mediated mRNA decay factor), *SLC16A2* (solute carrier family 16-member 2), and *SOGA1* (suppressor of glucose, autophagy-associated 1), with target scores above 98. Other significant targets include *EPPK1* (epiplakin 1), *WNT9B* (Wnt family member 9B), and *SOX13* (SRY-box 13), which play roles in cellular adhesion, autophagy regulation, and developmental processes. Additionally, several protocadherins, such as *PCDHA8* (protocadherin alpha 8) and *PCDHA1* (protocadherin alpha 1), are predicted targets, suggesting a potential involvement in cell–cell adhesion and signaling pathways that may influence glucose homeostasis in T2DM (Table 1).

### 3.4. Network Analysis of miR-4428 and miR-185-5p Target Genes

**Figure 3:**
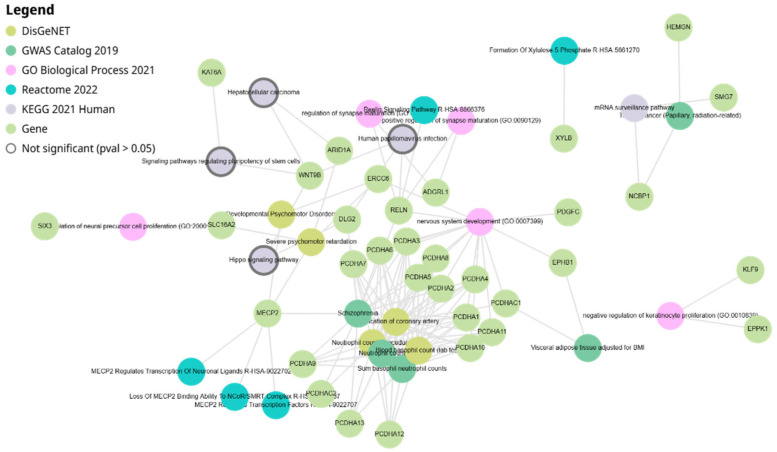
Network diagram illustrating the target genes of *miR-4428* and *miR-185-5p* and their associated biological pathways.

The network analysis of *miR-4428* and *miR-185-5p* target genes highlights intricate interactions among various genes and biological pathways. *miR-4428* and *miR-185-5p* regulate multiple cellular processes, including neuronal signaling, cell adhesion, and developmental pathways. Key target genes such as *MECP2*, *RELN*, and *KLF9* play crucial roles in neurodevelopmental processes, while others like *PCDH* clusters (e.g., *PCDHA1-13*) are associated with cell–cell adhesion and signaling mechanisms. Additional targets, including *WNT8B* and *ARID1A*, are linked to chromatin remodeling and signaling pathways. The network also illustrates connections to specific cellular functions like capillary surveillance and tissue development (Figure 3).

### 3.5. Enrichment Analysis of Biological Processes and Pathways

**Figure 4:**
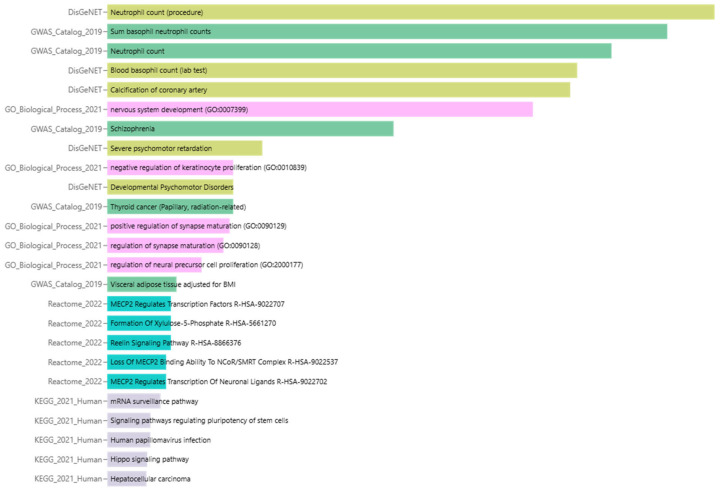
Enrichment analysis of biological processes and pathways associated with *miR-4428* and *miR-185-5p* target genes.

The enrichment analysis of *miR-4428* and *miR-185-5p* target genes reveals their significant involvement in diverse biological processes and pathways, emphasizing their potential roles in diabetes mellitus and associated conditions. Key enriched pathways include the regulation of synapse maturation and nervous system development (GO:007399), suggesting a connection to neuronal signaling and neurodevelopmental disorders such as schizophrenia and severe psychomotor retardation. Additionally, pathways like coronary artery calcification and visceral adipose tissue deposition highlight their involvement in cardiovascular and metabolic disorders. Specific processes such as *MECP2*-regulated transcriptional pathways, the Reelin signaling pathway, and chromatin remodeling mechanisms were identified, which may impact neuronal and cellular development. Genes linked to mRNA surveillance, the Hippo signaling pathway, and hepatocellular signaling further illustrate their functional relevance in broader cellular and tissue-level processes (Figure 4).

### 3.6. Summary of Enrichment Analysis Results

The enrichment analysis identified several significant terms associated with potential regulatory mechanisms in T2DM. The top-ranked terms included Neutrophil count (procedure) from DisGeNET, which showed an exceptionally low *p*-value of 8.11 × 10^−19^ and a combined score of 2917, highlighting its strong association with immune responses in T2DM. Additionally, the Sum basophil neutrophil counts and Neutrophil count from the GWAS Catalog 2019 were also significant, with *p*-values of 2.08 × 10^−17^ and 9.56 × 10^−16^, respectively, reflecting their involvement in immune cell regulation.

Several other biological processes, including blood basophil count (lab test) and calcification of the coronary artery, were significantly enriched in the DisGeNET library, with *p*-values of 1.01 × 10^−14^ and 1.63 × 10^−14^, respectively, indicating a possible link to cardiovascular complications in T2DM. In the Gene Ontology (GO) biological process category, terms such as nervous system development (GO:0007399) were also significantly enriched, with a *p*-value of 2.10× 10^−13^, suggesting a role in neuronal development and potentially in the neurovascular complications often seen in T2DM.

Other notable pathways include *MECP2* regulates transcription factors and Reelin signaling pathway from Reactome 2022, with a *p*-value of 0.01269 and a combined score of 435.5, which are implicated in neuronal signaling and may influence metabolic regulation. In contrast, pathways such as the mRNA surveillance pathway (KEGG 2021 Human) and signaling pathways regulating pluripotency of stem cells were also enriched but with higher *p*-values, indicating a more complex and broader set of regulatory mechanisms that may extend beyond insulin sensitivity (Table 2).

### 3.7. Assessment of miR-4428 and miR-185-5p Expression Profiles Across Human Organs Using miRNA Tissue Atlas 2025

**Figure 5:**
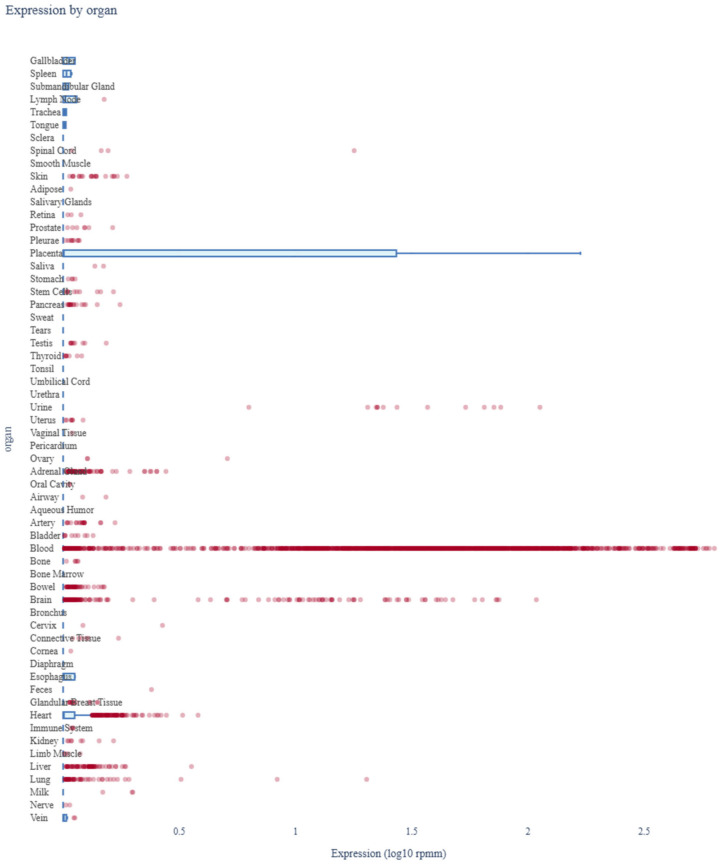
Expression profile of *miR-4428* across human organs. Red dots represent individual miRNA expression values, while blue bars indicate mean expression across tissue types.

The expression profile *miR-4428* across various human organs was analyzed using miRNA Tissue Atlas 2025 data. The analysis revealed that *miR-4428* is differentially expressed in multiple tissues, highlighting its potential role in regulating diverse biological processes. Notably, *miR-4428* showed significant expression in neuronal and cardiovascular tissues, suggesting its involvement in neurodevelopmental and cardiovascular functions. Additionally, its expression in metabolic and endocrine-related tissues supports its potential role in glucose metabolism and diabetes-related pathways (Figure 5).

**Figure 6:**
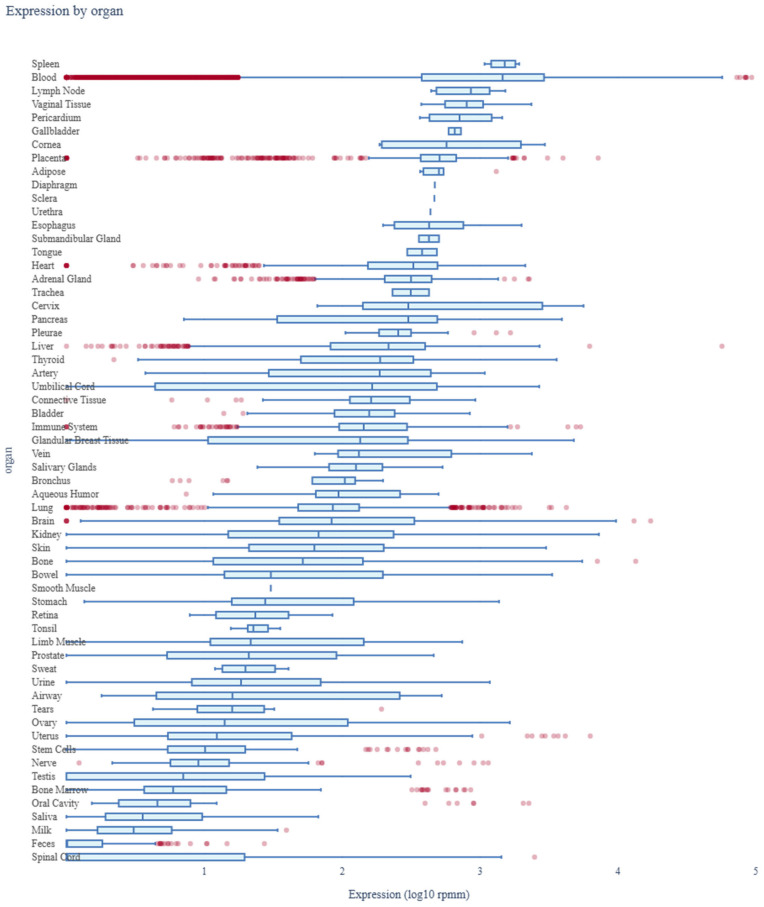
Expression profile of *miR-185-5p* across human organs. Red dots represent individual miRNA expression values, while blue bars indicate mean expression across tissue types.

The expression profile of *miR-185-5p* across various human organs was analyzed using data from the miRNA Tissue Atlas 2025. The analysis demonstrated that *miR-185-5p* is broadly expressed across multiple tissues, with notable expression in neuronal, metabolic, and cardiovascular tissues. Its significant presence in neuronal tissues suggests a potential role in neurodevelopmental processes and synaptic regulation. Additionally, the strong expression of *miR-185-5p* in metabolic and endocrine-related tissues supports its involvement in glucose metabolism and insulin signaling, indicating its relevance in the pathophysiology of diabetes mellitus (Figure 6).

## 4. Discussion

This study underscores the critical role of *miR-4428* and *miR-185-5p* in Type 2 Diabetes Mellitus (T2DM), particularly in insulin sensitivity and glucose homeostasis. The observed upregulation of *miR-4428* and significant downregulation of *miR-185-5p* in T2DM patients relative to controls highlight their potential as key regulators in metabolic dysfunction. These findings align with the emerging evidence linking microRNAs (miRNAs) to metabolic regulation and the pathophysiology of T2DM, emphasizing their role in post-transcriptional regulation of genes involved in glucose metabolism and insulin signaling [1,2].

The secondary structure analysis of *miR-4428* and *miR-185-5p* provides insights into their regulatory potential, suggesting that their unique stem–loop configurations facilitate specific interactions with target mRNAs, impacting stability and translational efficiency [3]. These structural features may underlie their functional roles in modulating key metabolic pathways.

Enrichment and target prediction analyses identified several key genes modulated by *miR-4428*, including *ADAR*, *KLF9*, *CCT8*, and *PDGFC*, all implicated in metabolic processes. *ADAR* plays a critical role in RNA editing, influencing metabolic gene expression, and its dysregulation may exacerbate insulin resistance [3]. Similarly, *KLF9*, a transcription factor, regulates gluconeogenesis and is linked to hyperglycemia under stress conditions, such as chronic inflammation in T2DM [4,5]. Furthermore, *miR-185-5p*’s predicted targets, including *SMG7*, *SLC16A2*, and *WNT9B*, suggest its involvement in cellular autophagy, glucose transport, and insulin signaling. The downregulation of *miR-185-5p* ob3-served in this study corroborates previous findings associating it with diminished glucose uptake and oxidative stress in T2DM [19,20].

The enriched pathways for *miR-4428* and *miR-185-5p* also provide a framework for understanding their roles in T2DM. Pathways such as the Reelin signaling pathway, which regulates neuronal plasticity, and the PI3K/Akt pathway, critical for insulin signaling, were notably associated with *miR-4428*. These findings suggest that *miR-4428* may have dual roles in metabolic and neurodegenerative processes, particularly given the links between T2DM and cognitive decline [9].

*miR-4428* has been implicated in neuronal signaling and chromatin remodeling, pathways that intersect with metabolic regulation. Its predicted targets, including *KLF9* and *ADAR*, suggest potential roles in glucose metabolism and insulin signaling. *KLF9* has been shown to influence gluconeogenesis, while *ADAR* plays a role in RNA editing, affecting metabolic gene expression. Similarly, *miR-185-5p* is predicted to target *SOGA1*, a key autophagy and glucose homeostasis regulator, and *WNT9B*, which has been linked to insulin sensitivity. These findings suggest that *miR-4428* and *miR-185-5p* may act as upstream regulators of metabolic pathways relevant to T2DM. Further studies are needed to validate these mechanistic roles experimentally.

The regulatory roles of *miR-4428* and *miR-185-5p* in glucose metabolism offer promising avenues for therapeutic intervention. Modulating their expression through targeted miRNA-based therapies could restore insulin sensitivity and mitigate hyperglycemia. The observed *miR-4428* upregulation, for instance, could be counteracted using miRNA inhibitors, while *miR-185-5p* mimics might enhance glucose transport and utilization.

Beyond T2DM, both *miR-4428* and *miR-185-5p* have demonstrated relevance in broader disease contexts. *miR-4428* is increasingly associated with cancer progression and metastasis. In lung adenocarcinoma (LUAD), it modulates the PI3K/AKT pathway via the circGRAMD1B/*miR-4428*/SOX4/MEX3A axis, enhancing migration, invasion, and EMT [16]. Conversely, it can act as a suppressor, as seen in LUAD, where interaction with ACTA2-AS1 promotes SOX7 expression and inhibits malignancy [21]. Its role in breast cancer as a potential serum biomarker for brain metastasis and its involvement in NSCLC progression through the LINC01806/miR-4428/NOTCH2 axis further underscore its multifaceted functionality [17,22]. Additionally, *miR-4428* is implicated in colonic and cervical neoplasms through regulatory interactions affecting *BCL2L11* and *PBX1* expression, respectively [23,24].

Similarly, *miR-185* has been implicated in metabolic and reproductive disorders. In diabetes mellitus (DM), particularly T2DM, *miR-185* expression is often reduced, coinciding with upregulation of NOS2, suggesting a role in inflammatory pathways [25]. Treatment with metformin has been shown to restore *miR-185-5p* expression, suppressing hepatic gluconeogenesis via inhibition of G6Pase [26]. Its plasma levels also correlate with T2DM progression [27]. Moreover, the downregulation of *miR-185* in serum and placenta has been associated with increased insulin resistance (HOMA-IR) in gestational diabetes mellitus (GDM), implying broader endocrine and metabolic regulatory roles [28].

The broader implications of *miR-4428* and *miR-185-5p* in various human diseases further support their potential roles as biomarkers and therapeutic targets, as detailed in Supplementary Table S2.

While this study primarily focused on identifying differential miRNA expression and associated pathways, Receiver Operating Characteristic (ROC) analysis would further validate the sensitivity and specificity of *miR-4428* and *miR-185-5p* as biomarkers. Future studies with larger independent cohorts will incorporate ROC analysis to confirm their clinical relevance.

While this study provides valuable insights into the differential expression of *miR-4428* and *miR-185-5p* in T2DM, the small sample size and reliance on bioinformatics-based analysis limit the statistical power of these findings. Although the FDR-corrected *p*-values for *miR-4428* and *miR-185-5p* exceeded conventional significance thresholds, these miRNAs were selected based on significant unadjusted *p*-values (*p* = 0.003 and *p* = 0.0072, respectively), strong fold changes, and known biological relevance. Future studies with larger cohorts and stricter multiple comparison corrections will help further validate these findings. Acknowledging the importance of functional validation in confirming the predicted roles of *miR-4428* and *miR-185-5p*, this study focused on bioinformatics-based predictions. Future research should include gain- and loss-of-function experiments, luciferase reporter assays, and CRISPR/Cas9 gene editing in pancreatic β-cells, hepatocytes, and adipocytes. Investigating their systemic effects, particularly in non-metabolic tissues, could further illuminate their roles in T2DM-associated complications.

## 5. Conclusions

This study identifies *miR-4428* and *miR-185-5p* as potential biomarkers and therapeutic targets in T2DM, highlighting their roles in insulin sensitivity and glucose metabolism. Pathway analyses link them to neuronal signaling, chromatin remodeling, and metabolic regulation. Their modulation may offer novel miRNA-based therapeutic strategies for improving insulin sensitivity. Future research should validate these findings and assess their clinical applicability in preclinical and clinical models.

## Figures and Tables

**Table 1:** Top predicted targets of *miR-4428* and *miR-185-5p* in regulating insulin sensitivity and glucose homeostasis in T2DM.

Target Rank	Target Score	Gene Symbol	Gene Description
Top Predicted Targets of *miR-4428*			
1	100	*EPHB1*	EPH receptor B1
2	99	*MECP2*	methyl-CpG binding protein 2
3	98	*KAT6A*	lysine acetyltransferase 6A
4	98	*ADAR*	adenosine deaminase, RNA specific
5	98	*RELN*	reelin
6	97	*CCT8*	chaperonin containing TCP1 subunit 8
7	97	*KLF9*	Kruppel like factor 9
8	96	*NCBP1*	nuclear cap binding protein subunit 1
9	95	*ST8SIA6*	ST8 alpha-*N*-acetyl-neuraminide alpha-2,8-sialyltransferase 6
10	95	*ZFP91*	ZFP91 zinc finger protein
11	95	*PDGFC*	platelet-derived growth factor C
12	95	*EGR3*	early growth response 3
13	95	*HEMGN*	hemogen
Top Predicted Targets of *miR-185-5p*			
1	99	*SMG7*	SMG7, nonsense-mediated mRNA decay factor
2	99	*SLC16A2*	solute carrier family 16-member 2
3	98	*SOGA1*	suppressor of glucose, autophagy associated 1
4	98	*EPPK1*	epiplakin 1
5	97	*WNT9B*	Wnt family member 9B
6	97	*SOX13*	SRY-box 13
7	97	*XYLB*	xylulokinase
8	97	*HP1BP3*	heterochromatin protein 1 binding protein 3
9	96	*PCDHA8*	protocadherin alpha 8
10	96	*PCDHAC1*	protocadherin alpha subfamily C, 1
11	96	*DLG2*	discs large MAGUK scaffold protein 2
12	96	*PCDHAC2*	protocadherin alpha subfamily C, 2
13	96	*PCDHA13*	protocadherin alpha 13
14	96	*ERCC6*	ERCC excision repair 6, chromatin remodeling factor
15	96	*PCDHA10*	protocadherin alpha 10
16	96	*PCDHA5*	protocadherin alpha 5
17	96	*ADGRL1*	adhesion G protein-coupled receptor L1
18	96	*PCDHA6*	protocadherin alpha 6
19	96	*PCDHA11*	protocadherin alpha 11
20	96	*PCDHA3*	protocadherin alpha 3
21	96	*PCDHA9*	protocadherin alpha 9
22	96	*ZNF704*	zinc finger protein 704
23	96	*RAE1*	ribonucleic acid export 1
24	96	*PCDHA4*	protocadherin alpha 4
25	96	*ABCG4*	ATP binding cassette subfamily G member 4
26	96	*PCDHA1*	protocadherin alpha 1
27	96	*PCDHA7*	protocadherin alpha 7
28	96	*PCDHA2*	protocadherin alpha 2
29	96	*CDH4*	cadherin 4
30	96	*CA10*	carbonic anhydrase 10
31	96	*PCDHA12*	protocadherin alpha 12
32	95	*TUBGCP3*	tubulin gamma complex associated protein 3
33	95	*FAM234B*	family with sequence similarity 234 member B
34	95	*ARID1A*	AT-rich interaction domain 1A
35	95	*C17orf77*	chromosome 17 open reading frame 77
36	95	*SIX3*	SIX homeobox 3
37	95	*RAB35*	*RAB35*, member RAS oncogene family
38	95	*ZNF236*	zinc finger protein 236

**Table 2:** Summary of the enrichment analysis results highlighting the significant biological processes and pathways associated with T2DM.

Term	Library	*p*-Value	q-Value	z-Score	Combined Score
Neutrophil count (procedure)	DisGeNET	8.11 × 10^−19^	8.71 × 10^−16^	70.02	2917
Sum basophil neutrophil counts	GWAS_Catalog_2019	2.08 × 10^−17^	2.52 × 10^−15^	53.4	2051
Neutrophil count	GWAS_Catalog_2019	9.56 × 10^−16^	5.79 × 10^−14^	38.88	1345
Blood basophil count (lab test)	DisGeNET	1.01 × 10^−14^	5.40 × 10^−12^	32	1031
Calcification of coronary artery	DisGeNET	1.63 × 10^−14^	5.82 × 10^−12^	22.67	719.8
nervous system development (GO:0007399)	GO_Biological_Process_2021	2.10 × 10^−13^	1.06 × 10^−10^	18.82	549.5
Schizophrenia	GWAS_Catalog_2019	2.97 × 10^−9^	1.20 × 10^−7^	9.171	180.1
Severe psychomotor retardation	DisGeNET	0.00002405	0.006457	27.3	290.3
Negative regulation of keratinocyte proliferation (GO:0010839)	GO_Biological_Process_2021	0.0001768	0.03812	135.7	1172
Developmental Psychomotor Disorders	DisGeNET	0.0001768	0.03164	135.7	1172
Thyroid cancer (Papillary, radiation-related)	GWAS_Catalog_2019	0.0001768	0.005347	135.7	1172
positive regulation of synapse maturation (GO:0090129)	GO_Biological_Process_2021	0.0002269	0.03812	116.3	975.7
regulation of synapse maturation (GO:0090128)	GO_Biological_Process_2021	0.0003455	0.04353	90.43	720.8
regulation of neural precursor cell proliferation (GO:2000177)	GO_Biological_Process_2021	0.001559	0.1179	38.73	250.4
Visceral adipose tissue adjusted for BMI	GWAS_Catalog_2019	0.008685	0.1631	15.32	72.72
*MECP2* Regulates Transcription Factors R-HSA-9022707	Reactome_2022	0.01269	0.3185	99.72	435.5
Formation Of Xylulose-5-Phosphate R-HSA-5661270	Reactome_2022	0.01269	0.3185	99.72	435.5
Reelin Signaling Pathway R-HSA-8866376	Reactome_2022	0.01269	0.3185	99.72	435.5
Loss Of *MECP2* Binding Ability To NCoR/SMRT Complex R-HSA-9022537	Reactome_2022	0.01772	0.3185	66.48	268.1
*MECP2* Regulates Transcription Of Neuronal Ligands R-HSA-9022702	Reactome_2022	0.01772	0.3185	66.48	268.1
mRNA surveillance pathway	KEGG_2021_Human	0.02593	0.4923	8.441	30.83
Signaling pathways regulating pluripotency of stem cells	KEGG_2021_Human	0.05155	0.4923	5.734	17
Human papillomavirus infection	KEGG_2021_Human	0.05225	0.4923	3.739	11.04
Hippo signaling pathway	KEGG_2021_Human	0.06493	0.4923	5.017	13.72
Hepatocellular carcinoma	KEGG_2021_Human	0.06844	0.4923	4.864	13.04

## Data Availability

All data supporting the results of this study are included within the article.

## Supplementary Materials

The following supporting information can be downloaded at: https://www.mdpi.com/article/10.3390/biology14040424/s1, Table S1. Demographic Data of Study Participants: Age, Gender, Fasting Blood Sugar (FBS), and HbA1c Levels for T2DM and Non-Diabetic Control Groups. Table S2. Associations between *miR-4428* and *miR-185* with human diseases [16,17,21,22,23,24,25,26,27,28,29].

## Abbreviations

The following abbreviations are used in this manuscript:

miRNA	MicroRNA
T1DM	Type 1 Diabetes Mellitus
T2DM	Type 2 Diabetes Mellitus
FBS	Fasting Blood Sugar
HbA1c	Hemoglobin A1c
lncRNA	Long Non-Coding RNA
FDR	False Discovery Rate
GO	Gene Ontology
β-cells	Beta cells
