# Supplementary material for: miR-4428 and miR-185-5p as Key Modulators of Insulin Sensitivity and Glucose Homeostasis: Insights into Pathways and Therapeutic Potential in Type 2 Diabetes Mellitus
Source: Biology (Basel). 2025 Apr 15;14(4):424. doi: 10.3390/biology14040424 (PMC12025167; doi:10.3390/biology14040424)
Supplement: Supplementary file 1 [file biology-14-00424-s001.zip › biology-3522211-supplementary.pdf]

biology

MDPI

Supplementary Table S1. Demographic Data of Study Participants: Age, Gender, Fasting Blood Sugar (FBS), and HbA1c Levels for T2DM and Non-Diabetic Control Groups.

No.	ID	Sex	Age	FBS (mg/dL)	HbA1c (%)
1.	DM1	M	33	270	13.8
2.	DM2	F	72	182	11.9
3.	DM3	M	83	205	11.7
4.	DM4	F	41	146	11.7
5.	DM5	F	40	145	11.3
6.	DM6	M	40	209	9.8
7.	DM7	F	62	191	9.8
8.	DM8	F	66	126	9.8
9.	DM9	F	62	211	9.2
10.	DM10	F	49	156	8.6
11.	NDM11	M	46	95	4.4
12.	NDM12	F	46	79	4.7
13.	NDM13	F	33	81	5.3
14.	NDM14	F	40	84	5.4
15.	NDM15	F	47	90	5.4
16.	NDM16	M	43	91	5.4
17.	NDM17	M	54	82	5.5
18.	NDM18	M	26	91	5.5
19.	NDM19	F	59	99	5.5
20.	NDM20	M	56	80	5.6

Supplementary Table S2. Associations between *miR-4428* and *miR-185* with human diseases.

Disease	Description	Category	Reference
**Associations Between *miR-4428* with Human Diseases**			
Adenocarcinoma of Lung	circGRAMD1B modulates the *miR-4428*/SOX4/MEX3A axis, activating the PI3K/AKT pathway and promoting migration, invasion, and EMT in LUAD cells.	other	21
Adenocarcinoma of Lung	ACTA2-AS1 inhibits LUAD cell malignancy by sequestering *miR-378a-3p* and *miR-4428*, enhancing SOX7 expression.	lncRNA target	22
Breast Neoplasms	Serum *miR-4428* and *miR-4480* could serve as biomarkers for predicting brain metastasis in breast cancer patients.	circulation_biomarker_diagnosis_NS	23
Carcinoma, Non-Small-Cell Lung	*In vitro* and *in vivo* assays confirmed that LINC01806 promotes NSCLC development through the *miR-4428*/NOTCH2 pathway . LINC01806 enhances NOTCH2 expression by sponging *miR-4428*, activating Notch signaling, and driving NSCLC progression, offering a novel target for therapeutic approaches.	lncRNA target	24
Colonic Neoplasms	LncRNA ACTA2-AS1 suppresses colon adenocarcinoma progression by sponging *miR-4428* and upregulating BCL2L11.	target gene	25
Uterine Cervical Neoplasms	RGMB-AS1 regulates the *miR-4428*/PBX1 axis to promote cervical cancer development, suggesting that targeting RGMB-AS1 could be a promising therapeutic strategy for cervical cancer patients.	lncRNA target	26
**Associations Between *miR-185* with Human Diseases**			

Disease	Description	Category	Reference
Diabetes Mellitus	The expression of NOS2 was upregulated, and the expression of *miR-185* was downregulated in the blood of patients with diabetes.	circulation_biomarker_ diagnosis_up	27
Diabetes Mellitus	The expression of NOS2 was upregulated, and the expression of *miR-185* was downregulated in the blood of patients with diabetes.	circulation_biomarker_ diagnosis_up	27
Diabetes Mellitus	Furthermore, metformin, an anti-diabetic drug, could upregulate *miR5p* expression to suppress G6Pase, leading to hepatic gluconeogenesis inhibition.	transcription factor target	28
Diabetes Mellitus, Type 2	5p in the plasma may be closely associated with the pathogenesis of T2DM.	circulation_biomarker_ diagnosis_ns	29
Diabetes Mellitus, Type 2	CONCLUSIONS: The data demonstrated that LncRNA MEG3 aggravated palmitate-induced insulin resistance by regulating the miR5p/Egr2 axis, providing new insights into T2D therapeutic strategies.	other	31
Diabetes, Gestational	CONCLUSIONS: The down-regulation of *miR-185* expression in serum and placenta of pregnant women with GDM is negatively correlated with HOMA-IR, suggesting that the decrease of *miR-185* may play an important role in the occurrence and development of GDM.	circulation_biomarker_ diagnosis_up	30
